# A Transition Metal‐Free Approach for the Conversion of Real‐Life Cellulose‐Based Biomass into Formate

**DOI:** 10.1002/advs.202415339

**Published:** 2025-03-31

**Authors:** Tong Zhang, Peng Ren, Yuman Qin, Thanh Huyen Vuong, Ana V. Cunha, Remco W. A. Havenith, Jabor Rabeah, Shoubhik Das

**Affiliations:** ^1^ Department of Chemistry University of Antwerp Antwerp 2020 Belgium; ^2^ Department of Chemistry University of Bayreuth 95447 Bayreuth Germany; ^3^ Leibniz‐Institut für Katalyse e.V. (LIKAT) 18059 Rostock Germany; ^4^ Stratingh Institute for Chemistry and Zernike Institute for Advanced Materials University of Groningen Groningen 9747 AG The Netherlands; ^5^ Ghent Quantum Chemistry Group Department of Chemistry Ghent University Gent 9000 Belgium; ^6^ State Key Laboratory of Low Carbon Catalysis and Carbon Dioxide Utilization Lanzhou Institute of Chemical Physics (LICP) Chinese Academy of Sciences Lanzhou 730000 P. R. China

**Keywords:** biomass transformation, formic acid, photocatalysis, upcycling

## Abstract

Formic acid (FA) and its salt are recognized as valuable molecules for various industries such as textiles and pharmaceuticals. Currently, the global demand of FA and its salts stands at 1.137 million metric tons per year, necessitating the development of sustainable methods to meet the future demands. While numerous approaches are developed for the generation of FA but the requirement of harsh reaction conditions to achieve them is unavoidable. On the other hand, the world production of biomass is estimated at 146 billion metric tons per year and that can be considered as a prospective source of FA and their salts. Additionally, cellulose accounts for approximately 35–45% of the biomass composition. Considering this, a visible‐light‐mediated approach is presented to produce formate directly from biomass at room temperature as well as at atmospheric pressure. In this approach, selective generation of hydroxyl radical has been achieved which later converted sugars, cellulose, and hemicellulose into formate. Furthermore, the conversion of cellulose‐rich daily‐life materials such as discarded paper into the product through a series of flow experiments is demonstrated. Finally, mechanistic investigations including electron paramagnetic resonance (EPR) spectroscopy, and density functional theory (DFT) calculations are conducted to gain insights into the underlying reaction mechanism.

## Graphical abstract

A visible‐light‐mediated method has been developed for the direct conversion of biomass into formate at ambient temperature and atmospheric pressure. This approach achieves the selective generation of hydroxyl radicals, which subsequently transform sugars, cellulose, and hemicellulose into formate. Additionally, the process has been applied to cellulose‐rich everyday materials, such as waste paper, demonstrating successful conversion through a series of flow experiments. To elucidate the underlying reaction mechanism, studies employing electron paramagnetic resonance (EPR) spectroscopy and density functional theory (DFT) calculations were conducted.

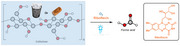

## 1 Introduction

**Figure 1:**
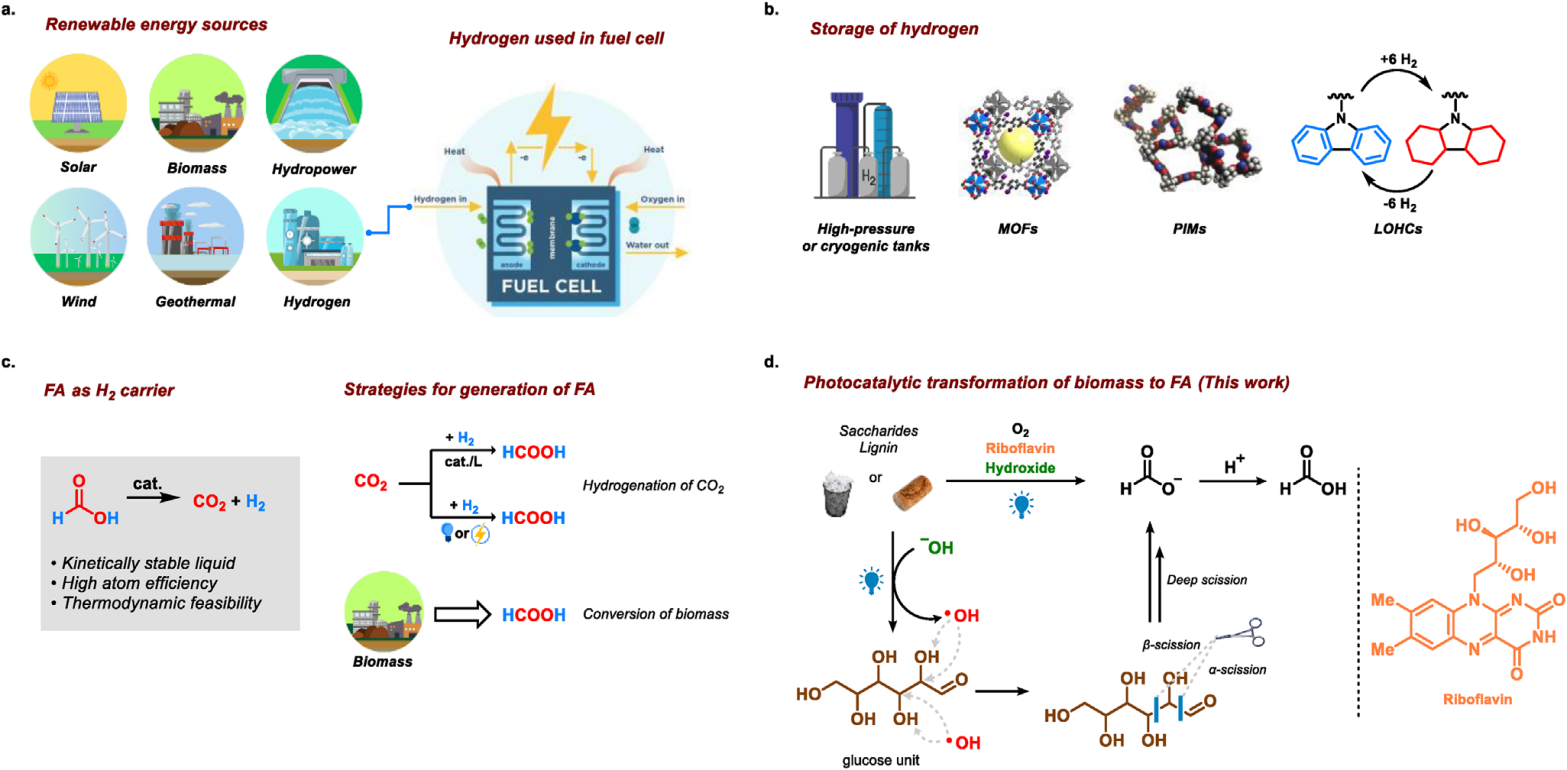
Formic acid as storage carrier of valuable hydrogen and strategies for photocatalytic transformation of biomass to formic acid.

Hydrogen, as an energy carrier, has become tremendously attractive since it can be employed within fuel cells to produce electricity, and power while generating water as the sole by‐product.^[^ ^1^, ^2^, ^3^, ^4^ ^]^ This has triggered hydrogen to be used in the domains of transportation and utilities as emerging sectors.^[^ ^5^ ^]^ Nevertheless, in order to expedite this approach, stringent efforts are required to address the storage and transportation challenges which are associated with hydrogen (**Figure** **1**a).^[^ ^6^ ^]^ Currently, significant discussions are ongoing for chemical and physical storage of hydrogen and among them, the most widely explored methods are involved by the use of high‐pressure tanks (with pressures reaching 700 bar) or cryogenic tanks (maintaining temperatures as low as −253 °C).^[^ ^7^ ^]^ Alternative approaches by employing low‐temperature adsorption and desorption processes on materials possessing substantial internal surface areas such as metal‐organic frameworks (MOFs), polymers of intrinsic microporosity (PIMs), zeolites, and carbon nanotubes are also under rapid investigations.^[^ ^8^ ^]^ In addition, organic compounds such as carbazole and others as viable hydrogen storage materials, have become an intriguing research direction (Figure 1b).^[^ ^9^ ^]^

In this aspect formic acid (FA) as a kinetically stable liquid at room temperature has garnered significant attention for hydrogen storage.^[^ ^10^ ^]^ Significantly, the utilization of FA demonstrates high atom efficiency due to the complete accessibility of all stored hydrogen for catalytic storage and dehydrogenation (DH) to H_2_.^[^ ^11^ ^]^ Additionally, formic acid and formate have been proven to be among the most promising fuels for direct liquid fuel cell.^[^ ^12^ ^]^ Furthermore, the thermodynamic feasibility of the conversion to CO_2_ is evidenced by a negative standard Gibbs free energy change (ΔG°) of −32.9 kJ mol^−1^ at ambient temperature.^[^ ^10^ ^]^ Moreover, FA as a valuable chemical finds widespread applications across diverse sectors such as pharmaceuticals, textiles, food, and agriculture. Furthermore, the sodium salt of formic acid (sodium formate) is often used as a de‐icer for runways and roads in continental climate zones, as a dyeing agent for printing or as a bleaching agent for textile and fabric applications.^[^ ^13^ ^]^ Particularly, sodium formate is often used as a leather tanning agent in India and in Brazil where leather industries play an important role in the national economy.^[^ ^14^ ^]^ Considering all these, the present global demand necessitates an annual supply of 1.09 million metric tons of FA. Therefore, significant attention is readily required to develop sustainable methods to produce FA and its salt to meet this requirement.^[^ ^9^, ^10^, ^15^ ^]^

To meet this demand, the hydrogenation of CO_2_ into FA will certainly represent an attractive route, however, this process necessitates elevated temperature, pressure, and costly transition metal catalysts along with the associated ligands.^[^ ^16^ ^]^ To avoid high temperature and pressure for the CO_2_ reduction, the utilization of photocatalytic or electrocatalytic processes will be ideal, however, it's still a long way to achieve this target.^[^ ^17^ ^]^ On the other hand, currently, the world production of biomass is estimated at 146 billion metric tons a year and that could be considered as a prospective source of FA.^[^ ^18^ ^]^ Among this, cellulose accounts for approximately 35–45% of the biomass composition. Indeed, the production of FA from biomass, such as cellulose, through processes like hydrolysis, catalytic oxidation, and electrocatalysis has been explored.^[^ ^14^ ^]^ However, these approaches are inadequate to meet high quantity of FA and are hindered by the associated challenges such as high energy consumption, elevated operational expenses, or stringent operational requirements (Figure 1c).^[^ ^19^ ^]^

Recently, photocatalysis has been successfully implemented for the conversion of carbohydrate‐based biomass into FA.^[^ ^20^ ^]^ These photocatalytic systems predominantly utilized titanium dioxide (TiO_2_) as the photocatalyst, necessitating activation by ultraviolet (UV) light which possesses significant health risks, including the potential for photooxidative damage to the retina. Additionally, its use can result in the formation of undesired by‐products, thereby reducing the selectivity of the intended reaction.^[^ ^21^, ^22^ ^]^ Therefore, considering the high selectivity and the necessity for low‐energy‐driven photoredox catalysis, such as that facilitated by visible light‐mediated photoredox processes, efficient conversion of cellulose‐based biomass into FA will hold strong potential for the contribution to the enhancement of sustainable approach around the globe.^[^ ^23^, ^24^ ^]^ In 2017, the Jin group conducted an investigation into the catalytic capabilities of various metal oxides, including nano‐TiO₂, zinc oxide (ZnO), and ferric oxide (Fe₂O₃), for the photochemical conversion of glucose into formate, employing a 125 W high‐pressure mercury lamp as the irradiation source.^[^ ^22a^ ^]^ While the production of formate was successfully achieved, the highest selectivity, at 35%, requires further optimization.

Given the considerable biomass conversion potential and the robust demand of FA, their integration presents innovative avenues for the sustainable production of FA. Herein, we have developed a visible light‐mediated transformation of biomass into FA by using riboflavin as a metal‐free photocatalyst at ambient temperature and pressure (Figure 1d). Riboflavin, a naturally occurring, biocompatible, and cost‐effective compound, demonstrates remarkable absorption of visible light and versatile redox properties, making it an ideal candidate for sustainable photocatalytic processes.^[^ ^26^ ^]^ Inspired by previous work for the cleavage of C─C bonds within glucose to form FA, the hydroxyl radical (·OH) emerged as a prime candidate for glucose degradation.^[^ ^27^ ^]^ This radical is one of the most potent oxidizing agents, thereby facilitating interactions with diverse chemicals such as bacteria, organic, and inorganic compounds.^[^ ^21^ ^]^ However, current photocatalytic strategies for hydroxyl radical generation typically necessitate harsh conditions, such as high‐intensity UV irradiation, which limits the applicability in broader fields and restricts industrial applications.^[^ ^22a^ ^]^ In this study, ·OH are efficiently generated under the irradiation with a 12 W household blue LED. These radicals abstract hydrogen atoms from glucose or biomass, subsequently inducing the cleavage of C─C bonds, ultimately leading to the production of FA.^[^ ^28^, ^29^ ^]^

## 2 Results and Discussion

Given that cellulose is a polysaccharide comprised of several hundreds to many thousands glucose units, the selection of glucose as a model substrate was undertaken to substantiate our conceptual framework (**Table** **1**).^[^ ^25^ ^]^ Initially, riboflavin was utilized as a photocatalyst in a solution of glucose and *n*‐butanol in the presence of a base under an O_2_ atmosphere. As shown in Table 1, FA was obtained in 40% yield within 20 h under the irradiation of 12 W household blue LEDs (entry 1). To verify the requirement of each component, a series of control experiments were also conducted and as expected, the absence of a light source and the base precluded the formation of FA, given that a light source and base were indispensable for the generation of hydroxyl radical (entries 2−6). In addition, oxygen as an oxidant was also essential in the system for closing the photocatalytic cycle. Furthermore, we observed that trace amount of FA was obtained even in the absence of the photocatalyst or glucose and the reason could be attributed to the alcohol oxidation process, followed by the C−C bond cleavage in the alcohol.^[^ ^30^ ^]^ In order to mitigate the excessive FA generation from alcohol, pure water was employed as a solvent and to our delight, an enhanced FA production rate of up to 45% was observed (entry 7). Additionally, more powerful light sources such as Kessil lamp with high intensity were also investigated to increase the yield, however, no improvement was obtained by adjusting the lights (entries 8−11). Therefore, 12 W household blue LED was the optimal light source for this transformation. In response to the growing demand for the sustainable reaction conditions, reducing the usage of a base is consistently considered as a favorable objective, provided that the reaction system remains unaffected. In pursuit of this goal, investigations were also conducted by using lower concentration of sodium hydroxide (NaOH) and the result revealed a clear trend: as the concentration of the base decreased, the yield of FA was also decreased (entries 12−14). Moreover, we expected higher yield of FA based on this trend, however, less FA was generated due to the limited solubility of sodium hydroxide in our reaction system (entries 15 and 16). Furthermore, we also explored rose bengal as a homogeneous photocatalyst under the optimized condition, however, it exhibited lower performances under our optimised reaction conditions or under the irradiation of upon irradiation with green light (entry 17 and 18), which could be due to its lower oxidation capacity in compared to riboflavin.^[^ ^31^ ^]^ Other well‐known organic photocatalysts, such as [Acr‐Mes]^+^ClO_4_^−^ (entry 19)and Eosin Y (entry 20) only produce the final product in very low yield under the optimal conditions.

**Table 1:** Optimization of reaction conditions for photocatalytic production of FA from glucose.

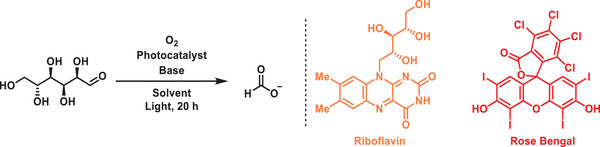					
Entry	Catalyst	Solvent (mL)	Base (M)	Light^a)^ (W)	Yield^b)^ (%)
1	Riboflavin	*n*‐Butanol (0.5)	NaOH (2.5)	12	40
2	Riboflavin	*n*‐Butanol (0.5)	NaOH (2.5)	Under dark	n.ob.
3	Riboflavin	*n*‐Butanol (0.5)	–	12	n.ob.
4^d)^	Riboflavin	*n*‐Butanol (0.5)	NaOH (2.5)	12	n.ob.
5	–	*n*‐Butanol (0.5)	NaOH (2.5)	12	8
6^e)^	Riboflavin	*n*‐Butanol (0.5)	NaOH (2.5)	12	4
7	Riboflavin	H_2_O (1)	NaOH (2.5)	12	45
8	Riboflavin	H_2_O (1)	NaOH (2.5)	24	43
9	Riboflavin	H_2_O (1)	NaOH (2.5)	40^c)^	43
10	Riboflavin	H_2_O (1)	NaOH (2.5)	20^c)^	26
11	Riboflavin	H_2_O (1)	NaOH (2.5)	10^c)^	29
12	Riboflavin	H_2_O (1)	NaOH (2)	12	43
13	Riboflavin	H_2_O (1)	NaOH (1.5)	12	40
14	Riboflavin	H_2_O (1)	NaOH (1)	12	35
15	Riboflavin	H_2_O (1)	NaOH (3)	12	33
16	Riboflavin	H_2_O (1)	NaOH (5)	12	17
17	Rose Bengal	H_2_O (1)	NaOH (2)	12	25
18^f)^	Rose Bengal	H_2_O (1)	NaOH (2)	12	13
19	[Acr‐Mes]^+^ClO_4_^−^	H_2_O (1)	NaOH (2)	12	20
20	Eosin Y	H_2_O (1)	NaOH (2)	12	15

Reaction conditions: glucose (0.2 mmol, 36 mg), photocatalyst (5 mol%), 20 h;

^a)^ Home‐made blue LED (456 nm);

^b)^ Formate was acidified to FA with 2 M HCl and yields were determined by ^1^H NMR with trimesic acid as internal standard;

^c)^ Kessil Lamp (456 nm);

^d)^ Under N_2_;

^e)^ No glucose;

^f)^ under irradiation of green light (550 nm).

**Figure 2:**
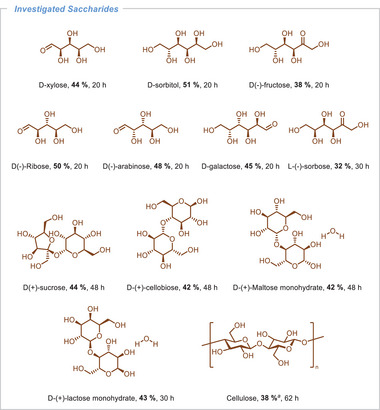
Photocatalytic production of FA from biomass. Formate was acidified to FA with 2 (M) HCl and yields were determined by ^1^H NMR with trimesic acid as internal standard. Cellulose was pretreated and dissolved in water by using freezing‐thawing method.

With the best reaction conditions in hand, we revisited the photocatalytic conversion of saccharides into FA (**Figure** **2**). Initially, the scope was expanded by systematically evaluating numerous monosaccharides, including D‐xylose, D(‐)‐fructose, D(‐)‐Ribose, D(‐)‐arabinose, D‐galactose and L‐(‐)‐sorbose, most of which can be obtained from natural products and biomass. It should be noted that all these saccharides underwent successful transformation into FA with moderate to good yields through the implementation of optimized photocatalytic reactions. Furthermore, various disaccharides including D(+)‐sucrose, D‐(+)‐cellobiose and D‐(+)‐Maltose monohydrate were also employed and demonstrated yields of 42 to 44%, however, in contrast to monosaccharide, the conversion of disaccharides necessitated longer reaction time. To move toward the main aim of this study, cellulose was also subjected in this investigation and revealed that even with an extended reaction time, the yield of FA was comparatively lower. The diminished photocatalytic performance was attributed to cellulose's limited solubility in water, which served as a crucial limiting factor.^[^ ^32^ ^]^ We must mention here that to dissolve the cellulose, various methods can be employed including using ionic liquid as a solvent, or via gasification or pyrolysis as well as pre‐treatment hydrolysis step.^[^ ^21^, ^33^ ^]^ However, these methodologies are associated with higher cost or stringent reaction conditions such as high temperature or pressure. To our delight, within our reaction system, a significant improvement in cellulose solubility was achieved through a pre‐treatment by employing the freeze‐thaw method, resulting in a notable FA yield of 38% based on carbon content (Figure S1, Supporting Information).^[^ ^34^ ^]^ This method demonstrated efficacy under our reaction conditions, necessitating neither the utilization of costly ionic liquid as a solvent nor the imposition of high temperature and pressure.

In the realm of photocatalysis, the challenges of extended reaction times and scaling up processes pose notable obstacles. Owing to the limited depth of penetration of blue light within the reaction medium, there is a consequential reduction in the photocatalytic efficiency.^[^ ^35^ ^]^ This arises from a substantial reduction in the photon flux during large‐scale reactions, ultimately impacts the overall performance.^[^ ^35^ ^]^ Inspired by our previous work, to enhance the efficiency of the developed photosystems, it is plausible to explore the implementation of reactions conducted within a flow system.^[^ ^36^ ^]^ Following a systematic examination of the oxygen and sample rates within the flow setup, the optimized conditions for flow reactions were ultimately determined (Table S1, Supporting Information). In addition, to further increase the photocatalytic efficiency, two powerful 40W Kessil lamp were employed by replacing the household blue LEDs in our flow set up. Delightfully, polysaccharides, namely cellulose and xylan, were capable of generating FA with the production rates of 0.60 and 0.61 mmol h^−1^, respectively (**Table** **2**, entries 1 and 2). Furthermore, lignin, a group of intricate organic polymers, also provided FA under our conditions with the production rate of 0.37 mmol h^−1^ (Table 2, entry 3). Recently, the valorization of everyday waste materials has garnered significant interest.^[^ ^37^ ^]^ Typically, cellulose content varies from 70% to 95% in wastepaper and from 40% to 45% in oak plugs. Remarkably, using our strategy, the valorization of daily waste materials such as wastepaper and oak plugs demonstrated the capability to produce valuable FA at promising production rates through a well‐designed flow system (Table 2, entries 4 and 5).

**Table 2:** Evaluation of various biomass‐materials from daily life.

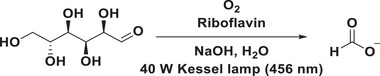				
Entry	Substrate	O_2_ flow rate (mL h^−1^)	Sample rate (mL h^−1^)	Production rate (mmol h^−1^)
1	Cellulose	1	0.3	0.60
2	Xylan	1	0.3	0.61
3	Lignin	1	0.3	0.37
4^a)^	Pre‐treated wastepapers	1	0.3	0.18
5^a)^	Pre‐treated Oak plugs	1	0.3	0.24

Reaction conditions: substrate (135 mg), riboflavin (56.4 mg), NaOH (2.0 M), H_2_O (15 mL), 2 lamp kessil (40W, 427nm). The volume of tube under the irradiation is 3.75 mL. Formate was acidified to FA with 2 (M) HCl and yields were determined by ^1^H NMR with trimesic acid as internal standard;

^a)^ Wastepapers and oak plugs were pretreated and dissolved in water by using freezing‐thawing method.

Inspired by all these outcomes, we became interested to investigate the reaction mechanism. To elucidate the mechanistic pathways, a series of quenching experiments were conducted (**Table** **3**). Specifically, radical scavengers, namely butylated hydroxytoluene (BHT) and (2,2,6,6‐tetramethylpiperidin‐1‐yl)oxyl (TEMPO), were introduced into the reaction system. These two radical scavengers effectively terminated reactions in which radical processes were implicated. Particularly, the addition of TEMPO significantly reduced the formation of FA, revealing the engagement of radical processes within the system. Additionally, the hydroxyl radical was successfully captured by using butylated hydroxytoluene (BHT) in a radical trapping experiment which clearly demonstrated the strong role of hydroxy radical in this process (Figure S4–S7, Supporting Information). However, all the attempts to detect the photogenerated hydroxy radical by using electron paramagnetic resonance (EPR) spectroscopy while using 5,5‐Dimethyl‐1‐pyrroline *N*‐oxide (DMPO) as a spin trap reagent and riboflavin as a homogeneous photocatalyst were failed, more probably due to its very short lifetime under the reaction conditions. Instead, the formation of superoxide radical as DMPO‐^·^O_2_ spin adduct was detected (Figure S9, Supporting Information). In spite of the higher stability of superoxide radical in alkaline solution, the EPR signal of DMPO‐^·^O_2_ spin adduct decayed within less than 40 s.^[^ ^38^ ^]^ This could also explain the reason why we were unable to detect the more reactive hydroxyl radical. In case of using rose bengal as a homogeneous photocatalyst, the formation of DMPO‐^·^OOH spin adduct was detected within less than 40 s (Figure S10, Supporting Information). This is in agreement with the low photostability and a slower rate of DMPO‐^·^OH adduct formation during irradiation in alkaline solutions.^[^ ^39^ ^]^

**Table 3:** Quenching experiments.

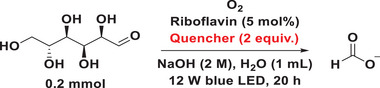			
Entry	Quencher	Be quenched	Yield (%)
1	TEMPO	Radical	25
2	9,10─Diphenylanthracene	^1^O_2_	49
3	CuCl_2_	SET	15
4	Benzoquinone	O_2_^·−^	22

Formate was acidified to FA with 2 M HCl and yields were determined by ^1^H NMR with trimesic acid as an internal standard.

Similarly, 9,10−diphenylanthracene and copper (II) chloride (CuCl_2_) were incorporated as scavengers for singlet oxygen and single electron transfer (SET) process, respectively. The results demonstrated that singlet oxygen adversely affected the production of FA, since slightly higher yield of FA was obtained by adding 9,10−diphenylanthracene. As expected, the addition of CuCl_2_ into the reaction mixture resulted in diminished yields, indicating the engagement of a SET process. Moreover, benzoquinone was used as a quencher for superoxide radical, leading to a reduced yield of FA. Additionally, fluorescence quenching experiment was conducted, revealing that the base exhibited the highest potential as a quencher for the excited state of the catalyst. Combing with the results of radical trapping experiments, it can be concluded that the base generated the key hydroxyl radical by interacting with the photocatalyst to catalyse the cleavage reaction (Figure S8, Supporting Information).

**Figure 3:**
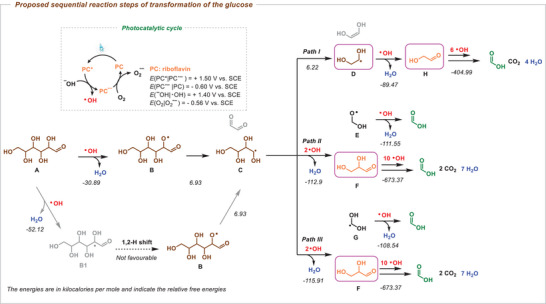
Proposed mechanism for sequential reaction steps of transformation of the glucose.

Typically, intermediates occurring during chemical reactions offer substantial insights conducive to mechanistic investigations.^[^ ^40^ ^]^ However, the direct identification of intermediates in the conversion of biomass into FA possesses considerable challenge.^[^ ^21f^ ^]^ This complexity arises due to the intricate nature of bond cleavage in biomass substrates, presenting a multitude of potential pathways and outcomes. Therefore, to investigate potential intermediates formed during the conversion of glucose to FA, diverse substrates were subjected to our experimental conditions (**Table** **4**).^[^ ^21^, ^41^ ^]^ Under the conditions of the model reaction, a substrate exhibiting a high yield would show a high possibility of serving as the intermediate. After investigating possible intermediates under our model reaction conditions, the results demonstrated that glycol, glycolaldehyde, and DL‐glyceraldehyde exhibited satisfactory yields of FA, albeit glycol exhibited lower conversion (Table 4). Conversely, other substrates such as glycolic acid, glyoxylic acid, and glyoxal achieved 100% conversion, albeit with lower production yields of FA. Furthermore, density functional theory (DFT) calculations substantiated the exothermic nature of the free Gibbs energies that are involved in the formation of glycol, glycolaldehyde, and DL‐glyceraldehyde, as well as the subsequent generation of FA (**Figure** **3**). Based on these findings, we hypothesize that glycol, glycolaldehyde, and DL‐glyceraldehyde are the most plausible intermediates in the transformation process.

**Table 4:** Identification of possible intermediates.

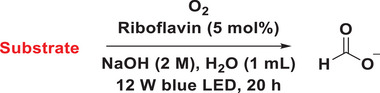			
Entry	Substrate	Conversion (%)^a)^	Yield (%)^b)^
1	Glycol	42	30
2	Glycolaldehyde dimer	100	33
3	Glycolic acid	cannot determine	14
4	Glyoxylic acid	100	19
5	Glyoxal	100	4
6	DL‐Glyceraldehyde	100	31
7	1,3‐Dihydroxyacetone	cannot determine	22

Reaction conditions: substrate (0.2 mmol), riboflavin (5 mol%), 20 h, NaOH (2 mM), H_2_O (1 mL), 12 W Home‐made blue LED (456 nm);

^a)^ Conversions of substrates were determined by 1H NMR with trimesic acid as internal standard;

^b)^ Formate was acidified to FA with 2 M HCl and yields were determined by 1H NMR with trimesic acid as internal standard.
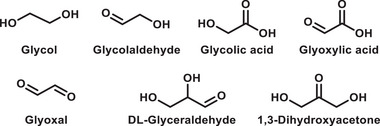

Based on all these investigations, we have presented the reaction steps for the conversion of glucose, elucidating its potential transformation pathways (Figure 3). At first, hydroxyl radical was generated via reductive quenching pathway, which was verified by the fluorescence quenching experiments. Later, the hydrogen atom of glucose **A** was extracted by the hydroxyl radical (·OH), leading to the formation of the oxygen‐centered radical **B**. It is worth noting here that the formation of radical **B1** is more thermodynamically favorable since the bond dissociation energy (BDE) of C−H bond is lower than that of O−H bond. Nevertheless, the subsequent steps for rationalizing the generation of essential intermediates and the formation of residue radicals or fragments from radical **B1** remain unattainable. In addition, the step for the transformation of **B1** to **B** via 1,2−hydrogen atom shift is not thermodynamically favorable. Therefore, we proposed that glucose **A** was first transformed into the radical **B** via a direct HAT process. Subsequently, a C−C bond cleavage occurred, resulting in the formation of the carbon‐centered radical **C** and glyoxal. Following the formation of **C**, three distinct pathways have been elucidated through mechanistic investigations: Path I involved the subsequent cleavage of radical **C**, resulting in the formation of radical **D**, which subsequently reacted with ·OH, leading to the production of one of the possible intermediates, glycolaldehyde (**H**). In the alternative pathway, radical **C** directly reacted with ·OH, leading to the formation of DL‐glyceraldehyde (**F**) along with either the oxygen‐centered radical (**E**) or the carbon‐centered radical (**G**), which underwent further reaction with ·OH to generate FA. Based on the analysis of the intermediate species (Table 4), it was postulated that compounds **D**, **F**, and **H** underwent subsequent reactions with ·OH, ultimately yielding the desired FA along with the by‐products CO_2_ and H_2_O.

## 3 Conclusion

In summary, we have developed a visible light‐mediated photocatalytic system for the transformation of real‐life cellulose‐based waste into valuable formic acid. Notably, our approach operates under mild reaction conditions, effectively circumventing the necessities of high temperature and high‐pressure that are commonly associated with such transformations. Furthermore, the implementation of a metal‐free photocatalyst, as opposed to noble metal catalysts such as palladium‐based catalysts, is employed for the reduction of the capital investment. Significantly, it is worth noting that daily waste materials, like wastepapers and oak plugs, can be effectively upcycled to yield high‐value products, aligning with the principles of green chemistry. We believe that our strategy will contribute to the transformation of biomass‐materials through visible light‐mediated photocatalysis and promote the advancement of supplementary methodologies in this field. Additionally, we have been also able to show that by the use of flow photocatalysis, reactions can be scaled up quite easily.

## Conflict of Interest

The authors declare no conflict of interest.

## Supporting information



Supporting Information

## Data Availability

The data that support the findings of this study are available from the corresponding author upon reasonable request.
