# Supplementary material for: A Transition Metal‐Free Approach for the Conversion of Real‐Life Cellulose‐Based Biomass into Formate
Source: Adv Sci (Weinh). 2025 Mar 31;12(21):2415339. doi: 10.1002/advs.202415339 (PMC12140328; doi:10.1002/advs.202415339)
Supplement: Supplementary file 1 — Supporting Information [file ADVS-12-2415339-s001.docx]

**Supporting** **information**

**A Transition Metal-free Approach for the Conversion of Real-life Cellulose-based Biomass into Formate**

Tong Zhang^‡a^, Peng Ren^‡a,b^, Yuman Qin^‡a,b^, Thanh Huyen Vuong^c^, Ana V. Cunha^a^, Remco W. A. Havenith^d^, Jabor Rabeah^e,c^, and Shoubhik Das*^a,b^

1. *Department of Chemistry, University of Antwerp, 2020 Antwerp, Belgium*
2. *Department of Chemistry, University of Bayreuth, 95447 Bayreuth, Germany*
3. *Leibniz-Institut für Katalyse e.V. (LIKAT), 18059 Rostock, Germany*
4. *Stratingh Institute for Chemistry and Zernike Institute for Advanced Materials, University of Groningen, Groningen 9747 AG, The Netherlands, and Ghent Quantum Chemistry Group, Department of Chemistry, Ghent University, 9000 Gent, Belgium*
5. *State Key Laboratory of Low Carbon Catalysis and Carbon Dioxide Utilization, Lanzhou Institute of Chemical Physics (LICP), Chinese Academy of Sciences, Lanzhou 730000, P. R. China*

^‡^ Denotes equal contributions

Corresponding authors: [shoubhik.das@uni-bayreuth.de](mailto:shoubhik.das@uni-bayreuth.de)

S1. Experimental section

## S1.1 Materials

NaOH, BHT (butylated hydroxytoluene), TEMPO (2,2,6,6-Tetramethylpiperidine-1-oxyl), 9,10-diphenylanthracene, CuCl_2_, Tert-butanol and Benzoquinone were purchased from Sigma Aldrich; DABCO (1,4-diazabicyclo [2.2.2] octane), Et_3_N (N, N-Diethylethanamine), Na_2_CO_3_, K_3_PO_4_ were purchased from TCI. Riboflavin was purchased from Alfa Aesar. Blue LED was home-made and the light intensity of LED (λ = 456 nm) was 0.0076 W/cm^2^. Kessil PR160-456nm lamp was purchased from Kessil^®^ with intensity 0.1 W/cm^2^ (2 cm distance).

## S1.2 Freeze-thaw method

Lignocellulose is Earth’s most abundant form of biomass, which is mainly comprised of cellulose (>40% in wood stems), surrounded by the less crystalline polymers hemicellulose and lignin. Among them, cellulose is a polymer that is widely found in nature and constitutes the main component of plant cell walls^1^. Due to its complex and highly bound structure, dissolving cellulose is not as easy as the normal dissolution process. Although, there are a number of methods that can be used to dissolve cellulose so that it forms a solution under specific conditions, such as dissolution of cellulose with ionic liquids^2^ and gasification, pyrolysis and pre-treatment hydrolysis/fragmentation steps^3^, it either needs very expensive ionic liquids as solvents or needs high temperature and high pressure, the most effective method is freeze-thaw treatment^4-5^:

Cellulose and stirring bar were put inside the 2M NaOH solution, then the suspensions were kept in a freezer at -20 °C for 4 h. Then, the formed frozen solids were thawed at room temperature with strong stirring. In the end, a clear and transparent cellulose solution was collected.


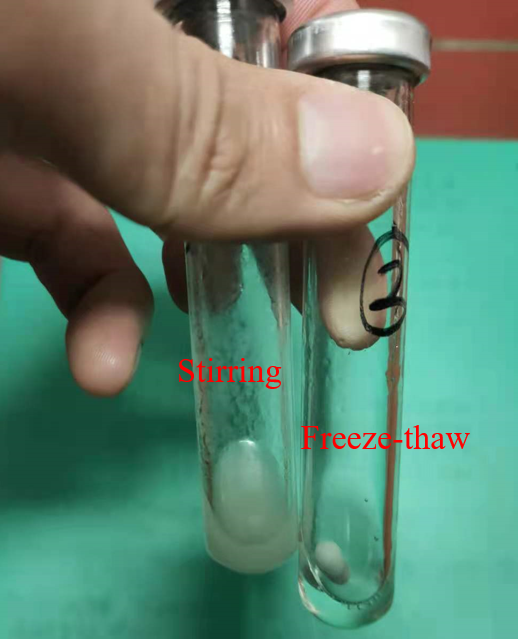


**Figure S 1**. Comparison of the dissolution effects of cellulose using ordinary stirring and freezing-thawing methods.

Other compounds of Lignocellulose, such as hemicellulose, also can be extracted by using freeze-thaw assisted alkali treatment.^6^

## S1.3 Photocatalytic experiments

### S1.3.1 Experimental procedure in batch reactor.

In a 28 mL schlenk tube containing glucose (36 mg), Photocatalyst Riboflavin (5 mol%, 3.76 mg) and stirring bar capped with rubber septum followed by vacuum (3 minutes) and O_2_ flow provided by O_2_ balloon (30 seconds) to make pure O_2_ atmosphere inside the tube. This process is repeated for three times. After that the O_2_ balloon was kept in this schlenk tube where needle is above on the solution, then1 mL 2M NaOH solution was added into the schlenk tube. At the end, the solution was irradiated at Home-made blue LED (12W, 456 nm) with vigorous stirring at 30^o^C temperature for 20 hr. The liquid product formate was first acidified to FA with 2 M HCl and then the yields were determined by ^1^H NMR with trimesic acid as internal standard.

To further verify the NMR peak of FA, commercially available FA was added to the sample after obtaining the crude NMR spectrum. As anticipated, no additional peaks appeared, while the intensity (or peak area) of the FA signals at identical chemical shifts increased.

Chemical shift of trimesic acid: 8.57 ppm, chemical shift of FA: 8.07 ppm.


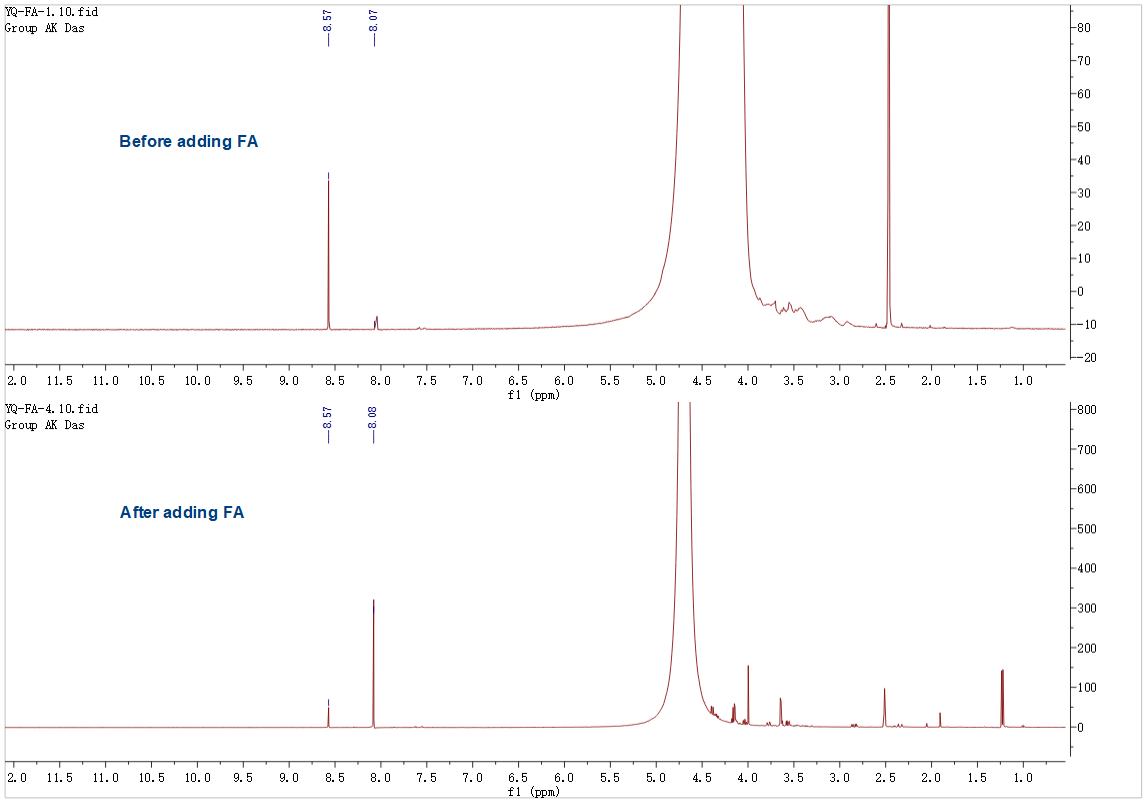


### S1.3.2 Experimental procedure in flow photoreactor.

In order to verify the industrial production potential of this method, we conducted large-scale experiment using flow setup.

In a 28 mL schlenk tube containing photocatalyst Riboflavin (56.4 mg) and stirring bar capped with rubber septum followed by vacuum (3 minutes) and O_2_ flow provided by O_2_ balloon (30 seconds) to make pure O_2_ atmosphere inside the tube. This process is repeated for three times. After that the O_2_ balloon was kept in this schlenk tube where needle is above on the solution, then cellulose solution (9mg/ml, 15mL) that prepared by using Freeze-thaw method was added into the schlenk tube.

First of all, in order to make pure O_2_ atmosphere inside the flow setup system, the O_2_ cylinder should be opened for around 20 minutes to flush the whole tubes by using a high O_2_ flow rate. After that, further investigations were conducted out in the flow photoreactor on a fixed O_2_ gas flow rate and sample flow rate respectively. At the light irradiation side, the solution was irradiated at 2 lamp kessil (40W, 427nm). For light set up contains two lights where two lamps were placed up and down and on either side of the reaction tube.

At the end, the liquid product formate was first acidified to formic acid with 2M HCl and then the yields were determined by ^1^H NMR with trimesic acid as internal standard.

## S1.4 Equipment Installation Diagram

### S1.4.1 Schematic representation of flow photoreactor system

**Figure S 2.** Schematic of flow reactor system.

### S1.4.2 Picture of flow photoreactor system


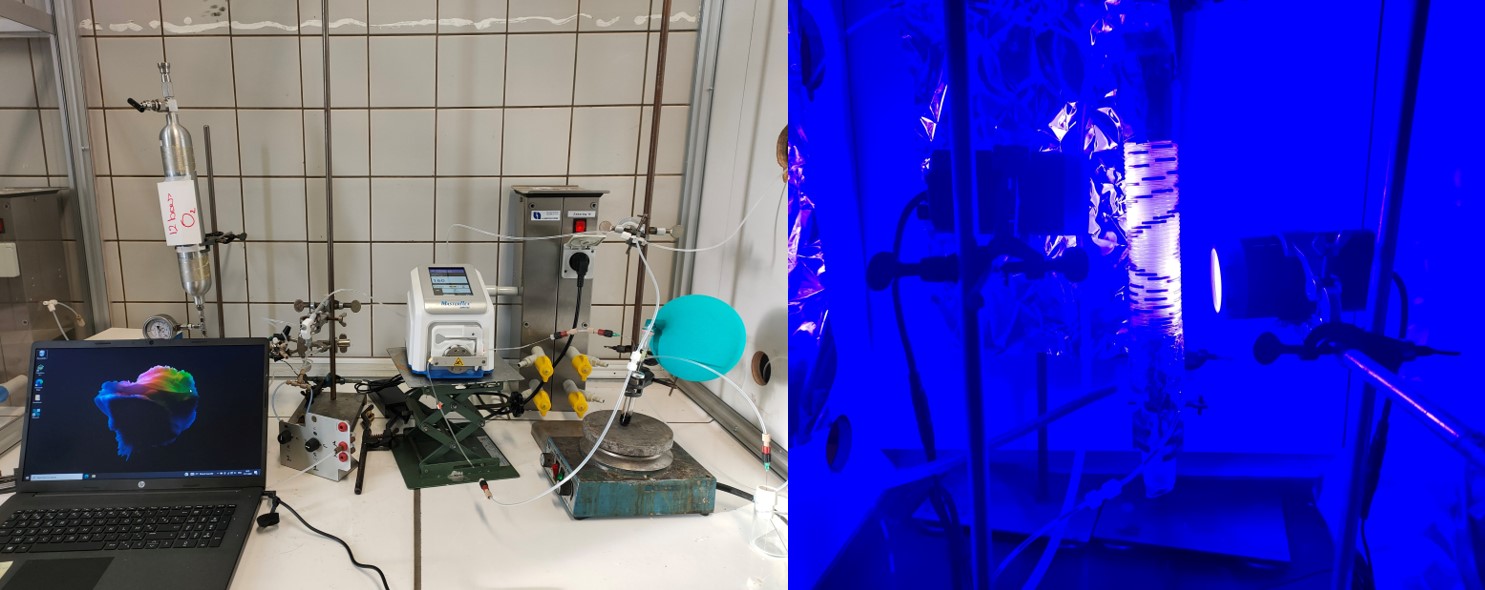


**Figure S 3.** Pictures of flow reactor system.

Specific parameters：

1. Total volume of reaction tube under irradiation is 4 mL
2. The distance between lamp and reactor is 3 cm.

## S1.5 Reaction conditions optimization in flow reactor system

**Table S 1:** Flow experiment optimization for cellulose oxidation to FA.

|  | | | | |
| --- | --- | --- | --- | --- |
| Entry | Amount of Substrate | O_2_ flow Rate | Sample flow rate | Yield % |
| 1 | 18 mg/mL | 5 mL/h | 3 mL/h | 13 |
| 2 | 18 mg/mL | 2 mL/h | 1 mL/h | 29 |
| 3 | 18 mg/mL | 1 mL/h | 0.5 mL/h | 32 |
| 4 | 9 mg/mL | 0.7 mL/h | 0.5 mL/h | 34 |
| 4 | 9 mg/mL | 1 mL/h | 0.5 mL/h | 43 |
| 5 | 9 mg/mL | 1 mL/h | 0.5 mL/h | 31 |
| **6** | **9 mg/mL** | **1 mL/h** | **0.3 mL/h** | **50** |
| 7 | 9 mg/mL | 1.25 mL/h | 0.25 mL/h | 40 |
| 8 | 9 mg/mL | 1 mL/h | 0.25 mL/h | 45 |
| 9 | 9 mg/mL | 0.75 mL/h | 0.25 mL/h | 42 |

Following a systematic examination of the oxygen and sample rates within the flow setup, the optimized conditions for flow reactions were ultimately determined as entry 6 in **Table S1**. Reaction conditions: substrate (135 mg), riboflavin (56.4 mg), NaOH (2.0 M), H_2_O (15 mL), 2 lamp kessil (40W, 427nm), 1 ml/h O_2_ flow rate and 0.3 ml/h sample flow rate.

# S2. Mechanism study

## S2.1 Radical trapping experiment

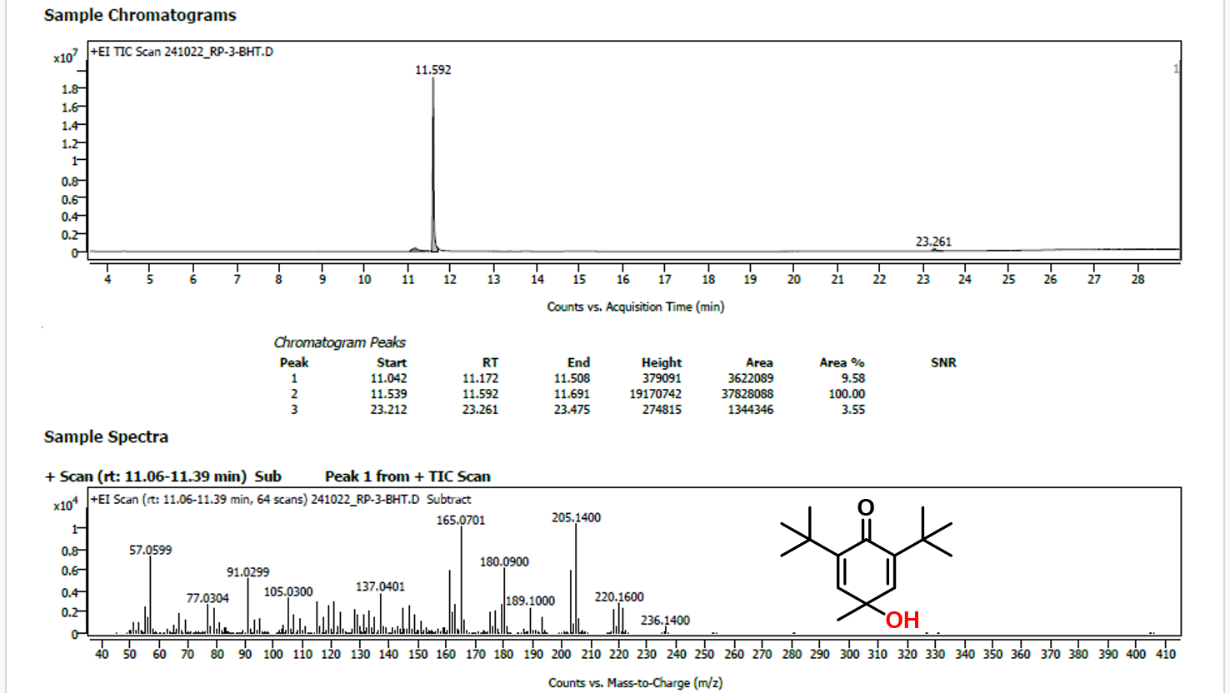


**Figure S 4** GC-MS data of radical trapping experiment conducted under reaction conditions with glucose.


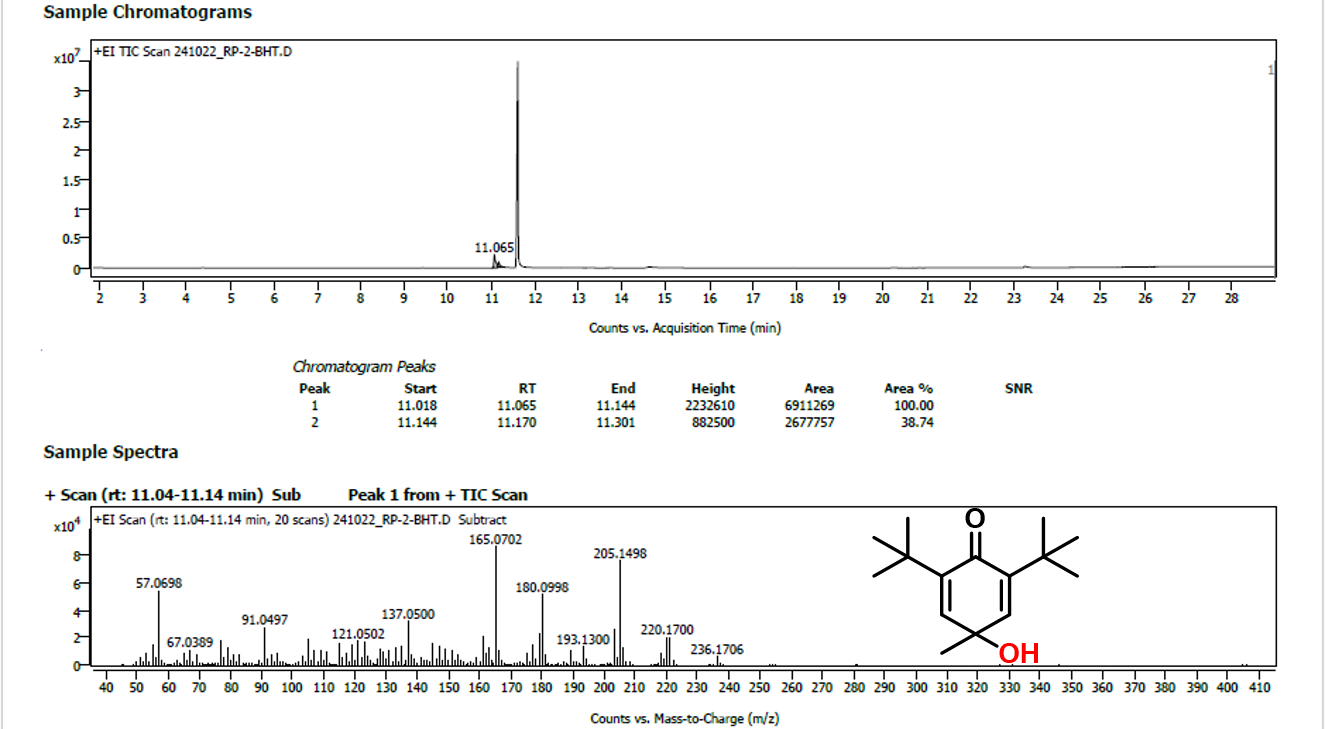


**Figure S 5** GC-MS data of radical trapping experiment conducted under reaction conditions without glucose.


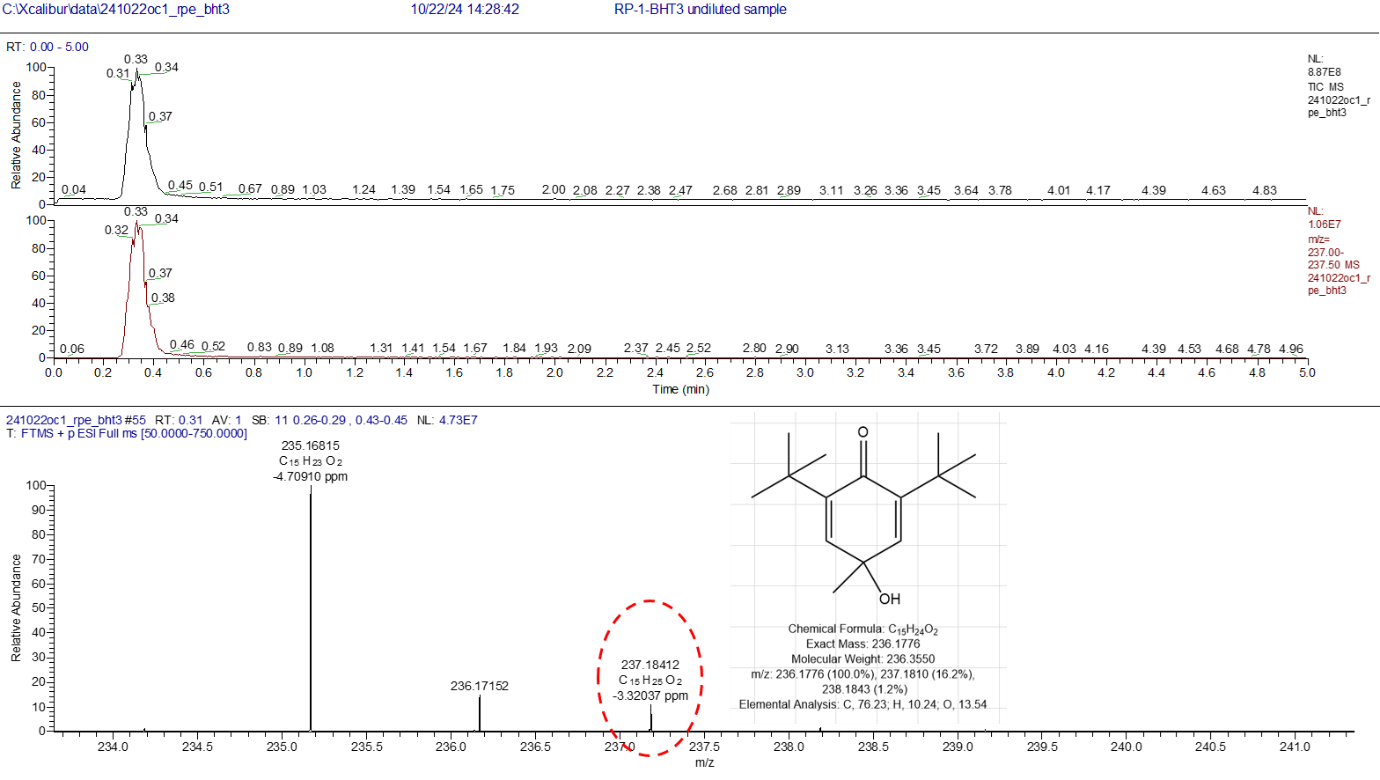


**Figure S 6** HRMS data of radical trapping experiment conducted under reaction conditions with glucose


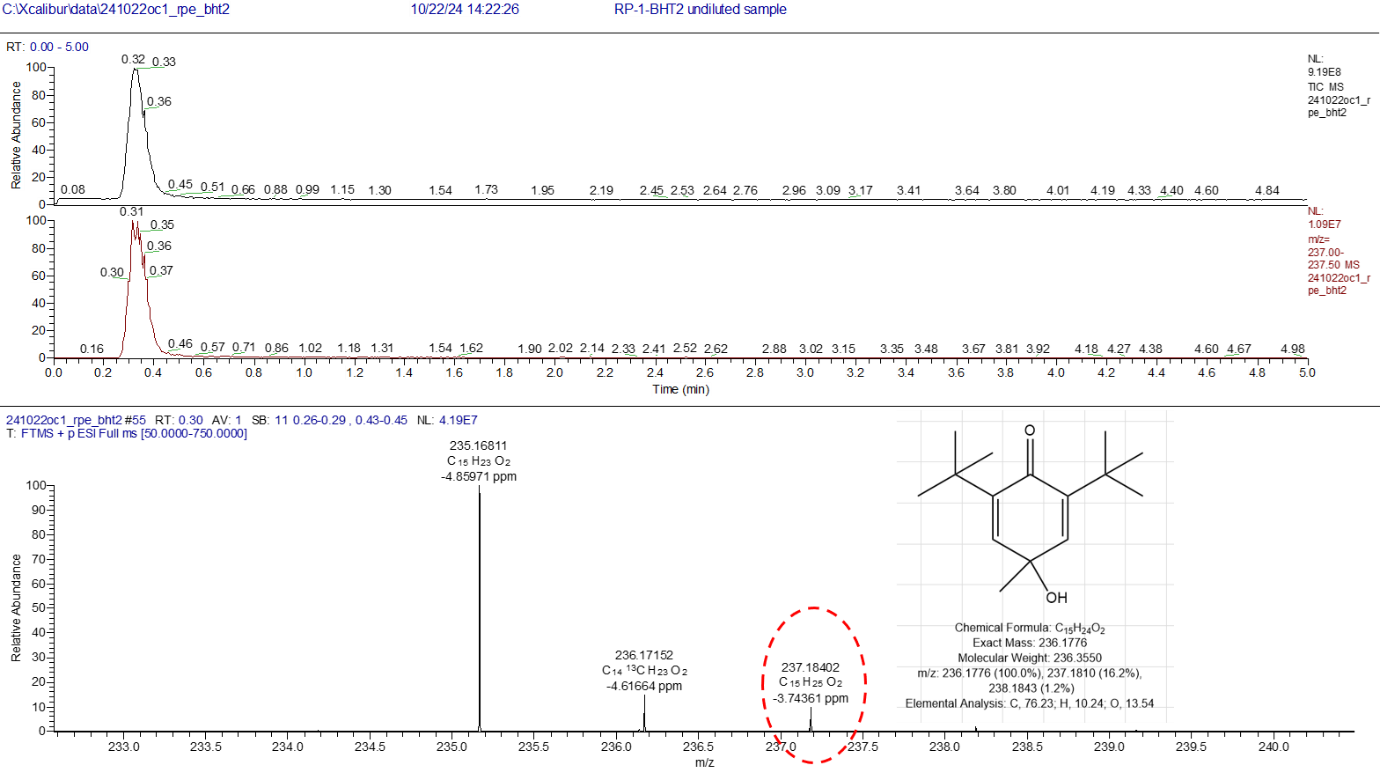


**Figure S 7** HRMS data of radical trapping experiment conducted under reaction conditions without glucose.

## S2.2 Stern-Volmer Plot (Fluoroscence Quenching Studies)

| 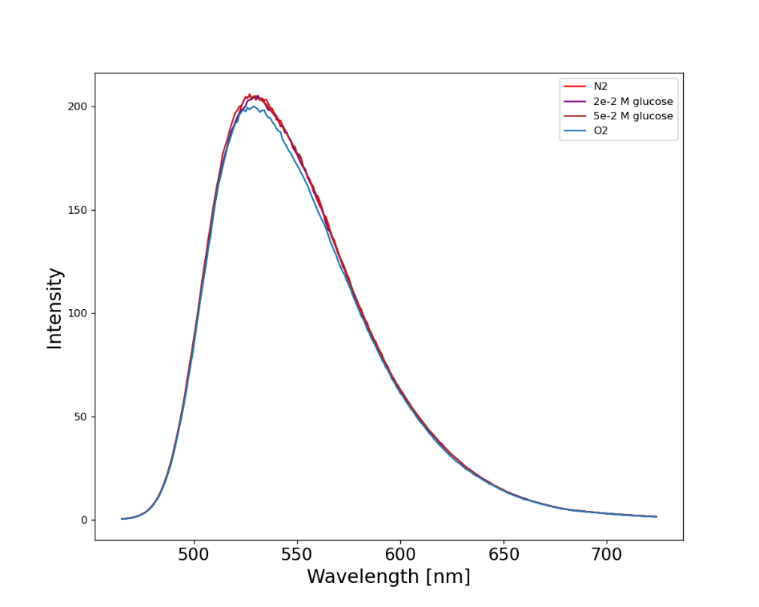 | 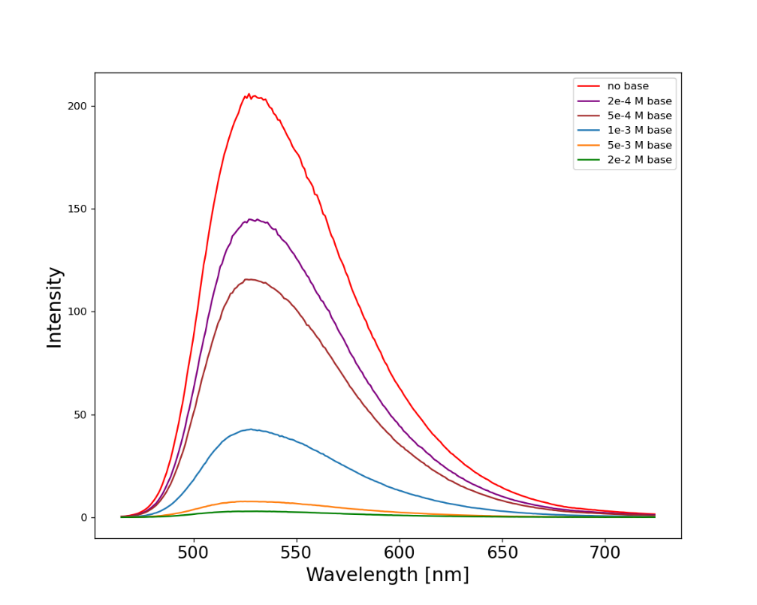 |
| --- | --- |

**Figure S 8.** Stern-Volmer Plot for Riboflavin with base, O_2_ and glucose as potential quenchers.

In the Fluorescence Quenching Studies, there is no decrease in the emission intensity with O_2_ or glucose, but the emission intensity decreases with the increase of the alkali concentration, it means Hydroxide anion rather than O_2_ or glucose is the first one to react with the excited state of the riboflavin catalyst and Hydroxide anion quenched the excited state of catalyst.

## S2.3 Evaluation of diverse bases and organic solvents.

**Table S 2.** *Evaluation of diverse bases with optimized conditions.*

|  | | | | |
| --- | --- | --- | --- | --- |
| Ent. | Substrate | Base (2M) | Time | yield% |
| 1 | Glucose | K_3_PO_4_ | 20h | 31 |
| 2 | Glucose | Na_2_CO_3_ | 20h | 5 |
| 3 | Glucose | Et_3_N | 20h | 21 |
| 4 | Glucose | DABCO | 20h | 4 |
| 5 | Glucose | NaOH | 20h | 43 |
| 6 | Formic acid | NaOH | 20h | 100 |

We can clarified from entries 1-5, the externally added hydroxide ions play a key role in biomass degradation and the resulting formate remains stable in this reaction system and is not futher converted (entry 6).

**Table S 3.** *Evaluation of diverse organic solvents with optimized conditions.*

|  | | | | |
| --- | --- | --- | --- | --- |
| Ent. | Substrate | Solvent (2M) | Time | yield% |
| 1 | Glucose | H_2_O | 20h | 43 |
| 2 | Glucose | MeCN | 20h | trace |
| 3 | Glucose | Acetone | 20h | trace |

## S 2.4 EPR spin trapping experiments with DMPO

EPR spectra were recorded on an X-band Bruker EMX CW-micro EPR spectrometer equipped with an ER4119HS high-sensitivity resonator using a microwave frequency of ν≈ 9.7 GHz in X-band, a microwave power of 6.3 mW, a modulation frequency of 100 kHz and a modulation amplitude of 1 G, a scanning number of 1 and sweeping time of 20 s. The hν = gβB_0_ equation was used to calculate g values with ν and B_0_ being the microwave frequency and resonance field, respectively. 2,2-Diphenyl-1-picrylhydrazyl (DPPH) was used as a standard (g = 2.0036 ± 0.0004) for calibration of the g value.

EPR spin trapping experiments with DMPO: The reaction mixture of riboflavin (1.4 mg) or rose bengal (3.6 mg), glucose (3.4 mg), NaOH (0.25M) (3 mL) was placed in a vial and sealed with a septum. O_2_ was bubbled by a syringe needle into this mixture at room temperature. About 100 μL of the reaction suspension was taken out and mixed with 10 μL DMPO. Then about 50 µL of this mixture was transferred into a glass microcapillary tube (Hirschmann) and EPR spectra were recorded in dark and under irradiation (40 W Kessil lamp 456nm) at room temperature. About 50 µL of reaction suspension without DMPO was also transferred into a glass microcapillary tube (Hirschmann) and EPR spectra were recorded under similar conditions.

_
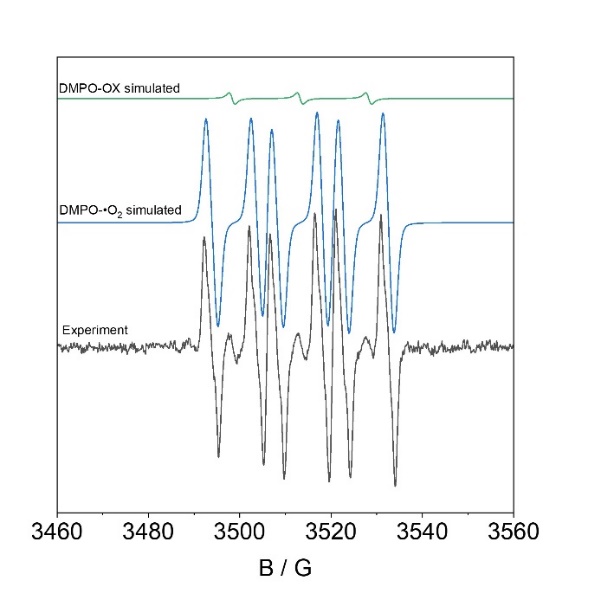
_

**Figure S 9.** Experimental and simulated EPR spectra of DMPO radical adducts formed after 40 sec irradiation of the reaction mixture of riboflavin (1.24 mM), glucose (6.3 mM) and DMPO (5 µL) in NaOH (0.25M) in the presence of O_2_, DMPO-^•^O2 simulated: a_N_ = 1.44 mT, a_H_^β^ = 1.00 mT, a_H_^γ^ = 0.12 mT; DMPO-OX simulated: a_N_ = 1.5 mT.

**
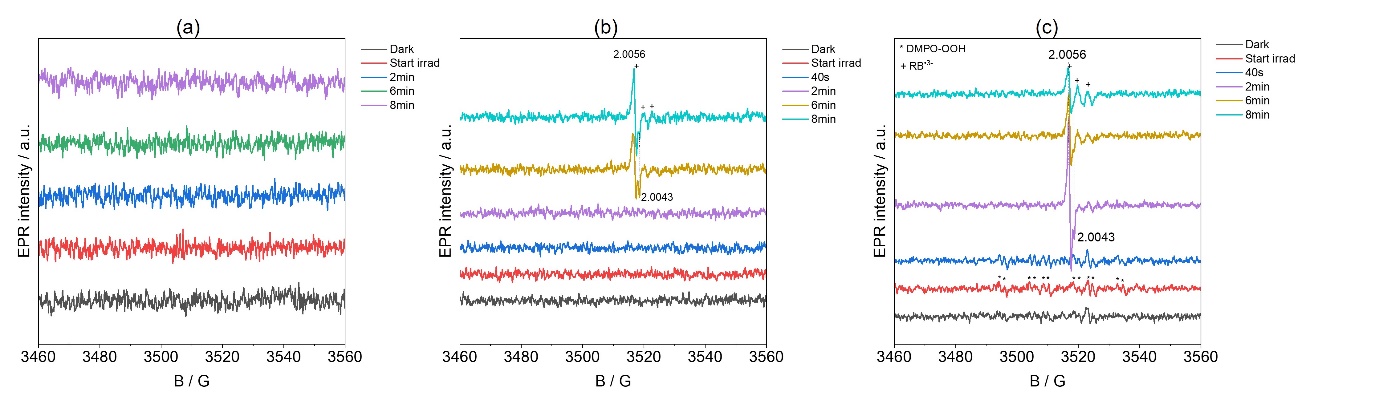
**

**Figure S 10.** EPR spectra of different reaction mixtures in the presence of O_2_ in dark and under the irradiation at room temperature (a) rose bengal (1.85 mM), glucose (10 mM) in H_2_O; (b) rose bengal (1.85 mM), glucose (10 mM) in NaOH (0.35M); (c) rose bengal (1.85 mM), glucose (10 mM), DMPO (5 µL) in NaOH (0.25M).

The EPR spectrum of different reaction mixtures in the presence of O_2_ using rose Bengal as a photocatalyst shown the EPR signal of O_2_^•-^ (g = 2.0056, 2.0043) after 6 min irradiation only in alkaline solution (Figure S 10b) but not in H_2_O (Figure S 10a), indicating the role of base in generation of hydroxyl radical and then superoxide radical to catalyse the reaction. Moreover, in the presence of DMPO as a spin trap reagent, the DMPO-OOH spin adduct was formed within very short time (Figure S 10c), following by the formation of O_2_^•-^ (g = 2.0056, 2.0043) together with the reduced rose bengal radical (RB^•3-^) (three-line signal with a_H_ = 3.2 G).^7-8^

## S 2.5 Active species of photocatalysis with HRMS analysis

Due to the potential instability and degradation of riboflavin under light irradiation in basic conditions, we investigated the active species of the photocatalyst. Initially, riboflavin was detected via HRMS after 5 hours, with the spectrum (**Figure S12**) matching that of pure riboflavin in H_2_O (**Figure S11**). However, after 7 hours, riboflavin was no longer detectable in HRMS (**Figure S13**). To identify the active species formed following riboflavin decomposition, HRMS analysis was conducted after the model reaction. The results confirmed the presence of lumichrome, a degradation product of riboflavin (**Figure S14**). To further validate this observation, lumichrome was employed as the sole photocatalyst in a model experiment, yielding approximately 17% of FA after 20 hours. Therefore, we conclude that the riboflavin serves as the initial active photocatalyst with a high catalytic efficiency. After 5 hours, riboflavin decomposes into lumichrome, which also functions as a reactive photocatalyst for the transformation, albeit with slower efficiency. Utilizing lumichrome as the sole photocatalyst results in a 17% yield of FA after 20 hours. This explains the necessity for a 20-hour reaction period to achieve optimal yield. During the initial 5 hours, riboflavin predominantly drives the transformation, while lumichrome assumes the primary catalytic role over the subsequent 17 hours.

**Figure S 11.** HRMS spectra of riboflavin in H_2_O.

**Figure S 12.** HRMS spectra of model reaction after 5 hours.

**Figure S 13.** HRMS spectra of model reaction after 7 hours. No peak of riboflavin can be found.


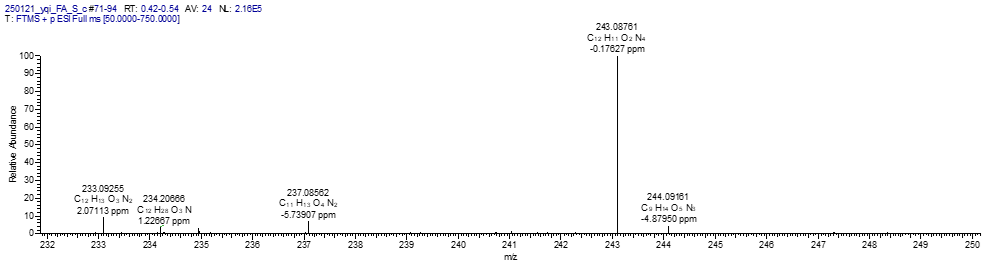


**Figure S 14.** HRMS spectra of model reaction after 24 hours.

# S3. Theoretical calculation

All calculations were performed with the AMS2022 suite.^9-11^ The PBE functional^12^ was chosen, combined with the TZ2P basis set^13-14^ (small frozen core). All geometries were optimized, and frequency calculations^15-16^ were performed to characterize the nature of the stationary point (no imaginary frequencies for minima, 1 imaginary frequency for transition states). The thermochemical data were calculated at T=298K, and P=1 atm. For radicals, unrestricted DFT calculations were performed, while for the closed-shell species restricted calculations were performed.

# Reference

1. D. W. Wakerley, M. F. Kuehnel, K. L. Orchard, K. H. Ly, T. E. Rosser, E. Reisner, *Nat. Energy* **2017**, *2*, 17021.
2. S. Zhu, Y. Wu, Q. Chen, Z. Yu, C. Wang, S. Jin, Y. Ding, G. Wu, *Green Chem.* **2006**, *8*, 325−327.
3. J. C. Colmenares, R. Luque, *Chem. Soc. Rev.* **2014**, *43*, 765−78.
4. N. Isobe, K. Noguchi, Y. Nishiyama, S. Kimura, M. Wada, S. Kuga, *Cellulose* **2013**, *20*, 97−103.
5. H. Qi, Q. Yang, L. Zhang, T. Liebert, T. Heinze, *Cellulose* **2010**, *18*, 237−245.
6. J. Li, Z. Liu, C. Feng, X. Liu, F. Qin, C. Liang, H. Bian, C. Qin, S. Yao, *Bioresour. Technol.* **2021**, *333*, 125107.
7. [P. Bilski](https://pubs.acs.org/action/doSearch?field1=Contrib&text1=P.++Bilski), [K. Reszka](https://pubs.acs.org/action/doSearch?field1=Contrib&text1=K.++Reszka), [M. Bilska](https://pubs.acs.org/action/doSearch?field1=Contrib&text1=M.++Bilska), [C. F. Chignell](https://pubs.acs.org/action/doSearch?field1=Contrib&text1=C.+F.++Chignell), *J. Am. Chem. Soc.* **1996**, *118*, 1330–1338.
8. J. L. Clément, [N. Ferré](https://scholar.google.com/citations?user=T-e2SFsAAAAJ&hl=zh-CN&oi=sra), D. Siri, H. Karoui, A. Rockenbauer, P. Tordo, *J. Org. Chem.* **2005**, *70*, 1198–1203.
9. E.J. Baerends, *ADF2022.01, SCM, Theoretical Chemistry, Vrije Universiteit, Amsterdam, The Netherlands,* [*http://www.scm.com*](http://www.scm.com), 2022.
10. C. Fonseca Guerra, J.G. Snijders, G. te Velde, E.J. Baerends, Towards an order-N DFT method. *Theor. Chem. Acc.* **1998,** *99*, 391−403.
11. G. te Velde, F.M. Bickelhaupt, E.J. Baerends, C. Fonseca Guerra, S.J.A. van Gisbergen, J.G. Snijders, T. Ziegler, Chemistry with ADF. *J. Comput. Chem.* **2001,** *22*, 931−967.
12. J.P. Perdew, K. Burke, M. Ernzerhof, Generalized Gradient Approximation Made Simple, *Phys. Rev. Lett.* **1996**, *77*, 3865−3868.
13. E. van Lenthe, E.J. Baerends, Optimized Slter-type basis sets for the elements 1-118, *J. Comput. Chem.* **2003**, *24*, 1142−1156.
14. D.P. Chong, E. van Lenthe, S.J.A. van Gisbergen, E.J. Baerends, Even-tempered slter-type orbitals revisited: From hydrogen to krypton, *J. Comput. Chem.* **2004**, *25*, 1030−1036.
15. S.K. Wolff, Analytical second derivatives in the Amsterdam density functional package, *Int. J. Quantum Chem.* **2005**, *104*, 645−659.
16. L. Fan, T, Ziegler, Application of density functional theory to infrared absorption intensity calculations on transition-metal carbonyls, *J. Phys. Chem.* **1992***, 96*, 6937−6941.
